# Self-Supervised Based Multi-View Graph Presentation Learning for Drug-Drug Interaction Prediction

**DOI:** 10.53941/tai.2025.100001

**Published:** 2024-10-23

**Authors:** Kuang Du, Jing Du, Zhi Wei

**Affiliations:** 1 Department of Computer Science, New Jersey Institute of Technology, Newark, NJ 07102, USA; 2 Department of Electrical and Computer Engineering, Rutgers University, Piscataway, NJ 08854, USA

**Keywords:** hierarchical graph representation learning, self-supervised learning, drug-drug interaction, molecular structural information

## Abstract

Drug-Drug Interactions (DDIs) can occur when diseases are treated with combinations of drugs, leading to changes in the pharmacological activity of these drugs. Predicting DDIs has become a crucial task in medical health. Recently, hierarchical graph representation learning methods have attracted significant interest and have proven effective for this task. However, collecting drug interaction data through biological experiments in wet laboratories is resource- and time-intensive. Given the limited amount of available drug interaction data, the performance of existing hierarchical graph methods has encountered a bottleneck. Current approaches are supervised learning methods, which train graph neural networks on specific datasets and can cause overfitting problems. Additionally, supervised learning models cannot leverage information from massive amounts of unlabeled public molecular datasets, such as ZINC15. To overcome this limitation, we propose a novel method for multi-view graph representation learning, namely, Self-Supervised Multi-View Graph Representation Learning for Drug-Drug Interaction Prediction (SMG-DDI). SMG-DDI leverages a pre-trained Graph Convolutional Network to generate inter-view molecule graph representations, incorporating atoms as nodes and chemical bonds as edges. Subsequently, SMG-DDI captures intra-view interactions between molecules. The final drug-drug interactions will be based on the drug embeddings from intra-view analyses. Our experiments conducted on various real datasets demonstrate that molecular structure information can aid in predicting potential drug-drug interactions, and our proposed approach outperforms state-of-the-art DDI prediction methods. The accuracies are 0.83, 0.79, and 0.73 on small, medium, and large scale test datasets, respectively.

## 1. Introduction

Drug-drug interactions (DDIs) refer to the phenomenon where two or more drugs, when combined, alter each other’s pharmacological effects [1]. These interactions can lead to various outcomes, either enhancing or inhibiting the efficacy of the drugs, increasing the risk of patient harm, or even prompting the withdrawal of drugs from the market. A recent study [2] by the U.S. Centers for Disease Control and Prevention on prescription drug use indicates that, among adults aged 40–79, approximately 1 in 5 use at least five prescription drugs simultaneously. Therefore, predicting DDIs in advance is of paramount importance in clinical practice.

The accurate identification of Drug-Drug Interaction (DDI) relationships traditionally relies on in vivo trials in medicine. In vitro trials offer an alternative, albeit limited, especially when dealing with numerous unstudied drugs or attempting to simulate challenging cellular environments, such as those found in bone and prostate cells [3]. Various machine-learning methods have emerged for DDI relationship detection. In the early stages of machine learning for DDI identification, similarity-based approaches were prevalent. These approaches included measuring 2D structural fingerprints [4], utilizing nearest neighbor algorithms for prediction, and employing logistic regression models with fingerprints as input. Then deep-learning approaches, such as DeepDTIs [5] and LASSO-DNN [6], were shown to have greater power than traditional machine learning algorithms. However, these methods have become less favored due to their labor-intensive feature extraction process. Instead more advanced graph models have gained popularity in the field [7]. In recent years, the exploration of graph representations has garnered increased attention, primarily propelled by advancements in graph neural networks (GNNs). These graph-based methods can broadly be categorized into two types: molecular graph-based models and hierarchical graph-based models. Molecular graph-based models exclusively leverage the structural features of drug molecules extracted by GNNs. Subsequently, the generated representations of drugs are employed to predict interactions between drug pairs [8,9]. While these molecular models have effectively addressed challenges related to feature engineering, yielding commendable results in DDI prediction tasks, they tend to overlook the vital topological information between drugs. Hierarchical graph-based models, exemplified by BI-GNN [10] and MIRACLE [11], have explored multi-view approaches by integrating both molecular and topological information. This integration provides a more comprehensive approach to drug interaction prediction. Experimental results indicate that hierarchical graph-based models outperform molecular graph models in DDI prediction. The limitation of hierarchical graph-based models for generalization lies in its reliance on labeled training data, which may not fully represent the diversity of real-world scenarios, potentially hindering the model’s ability to generalize accurately to unseen or novel inputs. To address the limitation of supervised learning, self-supervised learning provides a promising learning paradigm that reduces the dependence on manual labels [12]. The success of self-supervised learning techniques in Computer Vision and Natural Language Processing has prompted the popularity of pre-training methods in graph-related applications, particularly in molecular graph models where labeled data is scarce. Molecular-scale graph pre-training methods predominantly include generation-based [13], predictive-based [14]. A notable example is GraphMVP [15], which focuses on pre-training methods that leverage 3D geometric information to learning atom, bond, and molecular-level information. This approach translates to superior performance across various downstream tasks. Consequently, incorporating self-supervised learning in training molecular graph models shows significant promise enhance molecular graph representation.

Even though the above-mentioned hierarchical graph-based methods achieve satisfactory results and become the state-of-the-art in DDI prediction tasks, most of those methods, are experimenting on random splitting datasets. However, it is common to see the graph data from real-world applications often contain out-of-distribution samples [16], meaning that graphs in the training dataset are structurally very different from graphs in the test dataset. Random data splitting approach is not considering this situation. In many chemistry domain applications, the conventional random data splitting approach tends to be overly optimistic and fails to replicate real-world scenarios, where test graphs can exhibit structural differences from the training graphs [17,18]. As a result, using a random data splitting approach cannot fully validate the model’s generalization ability. In contrast, the scaffold data splitting method [19] categorizes molecules according to their scaffold (molecular substructure). The scaffold data splitting setting partitions the data based on two-dimensional structural frameworks, which include ring systems and linkers [20], providing a more realistic evaluation. Priori studies, Chen et al. [21] and Sheridan [22], have shown that scaffold data splitting provides a more realistic estimate of model performance in prospective evaluation compared to random data splitting approach. Researchers like Hu et al. [16] and Wu et al. [17] have successfully used scaffold data splitting for molecular benchmarks, emphasizing its effectiveness in assessing generalization capabilities.

**Figure 1.**
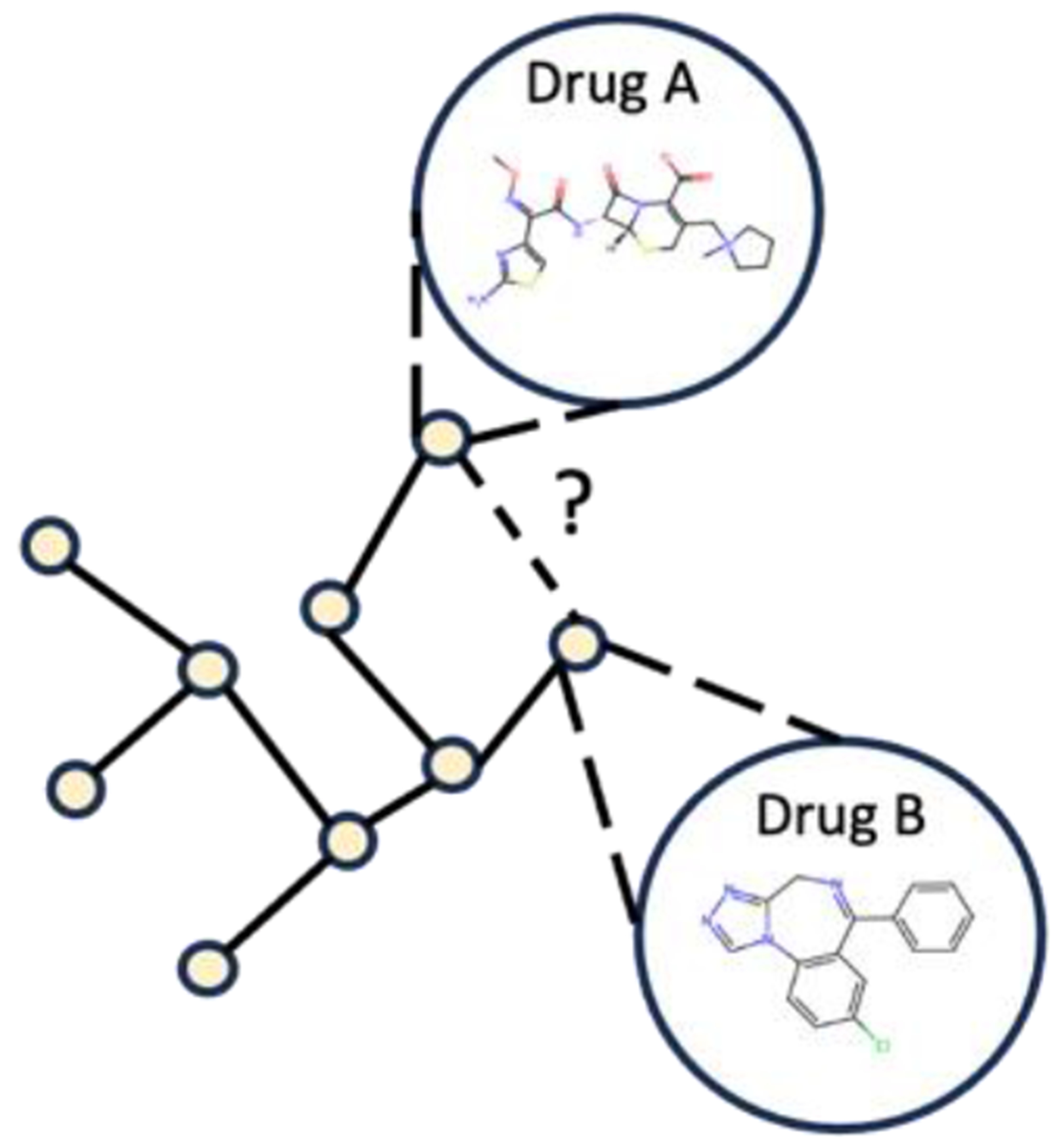
Multi-view graph of Drug-Drug Interaction.

Here we introduce a novel method for multi-view graph representation learning, named Self-supervised based Multiview Graph representation learning for Drug-Drug Interaction Prediction (SMG-DDI). Our approach involves two levels of graphs within the multi-view setting. The inter-view drug molecular graph represents drug instances, comprising atoms as nodes and chemical bonds as edges. The intra-view drug interaction network graph consists of drugs as nodes and external DDI relationships as edges. In the inter-view, we employ a pretrained graph convolution network for embedding drug molecular graphs. The drug-drug link predictor utilizes intra-view drug interaction graph embeddings to predict unknown interactions, essentially filling in the missing links between drugs. We employed a Central Moment Discrepancy (CMD) [23] regularization term to minimizes the distribution discrepancy between inter-view and intra-view graph representations. Figure 1 provides an illustration of the multi-view graph context. In the DDI network, drug A and B represent two drugs, while solid and dashed lines denote existing and potential interactions. The internal structure of each drug is depicted by its molecular graph.

Compared to hierarchical graph models such as SEAL-CI and MIRACLE, our experiments demonstrate that the SMG-DDI model improves DDI prediction. The key contributions of this work are as follows:

1. Self-Supervised Learning: Our SMG-DDI model adopts a self-supervised approach, leveraging the large, unlabeled molecular dataset ZINC15 to extract molecular features. This enhances model robustness and reduces overfitting. SMG-DDI incorporates three pretraining strategies—Context Prediction, Edge Prediction, and Masking Node Prediction—combined with GCN models. In contrast, SEAL-CI and MIRACLE rely on supervised learning, which is constrained by the need for labeled training data to learn graph representations.
2. Efficient Handling of Over-Smoothing: While MIRACLE mitigates over-smoothing through contrastive learning, this approach demands substantial computational resources, as it requires both negative and positive training datasets. Our model, however, reduces distribution mismatches between the hierarchical graph layers using space matching and feature space matching, making it computationally efficient. Coupled with self-supervised learning, SMG-DDI outperforms MIRACLE in both performance and resource efficiency.
3. Scaffold Data Splitting for Generalization: We propose using scaffold data splitting in DDI prediction tasks to better evaluate a model’s generalization across various public datasets. Unlike the random splitting strategies used by most baseline hierarchical graph models for drug-drug interaction prediction, our approach provides a more realistic evaluation of the model’s applicability in real-world scenarios.
4. Improved Performance: Through extensive experiments on multiple real-world datasets, we demonstrate that the SMG-DDI model achieves superior prediction performance compared to state-of-the-art methods.

## 2. Material and Methods

**Figure 2.**
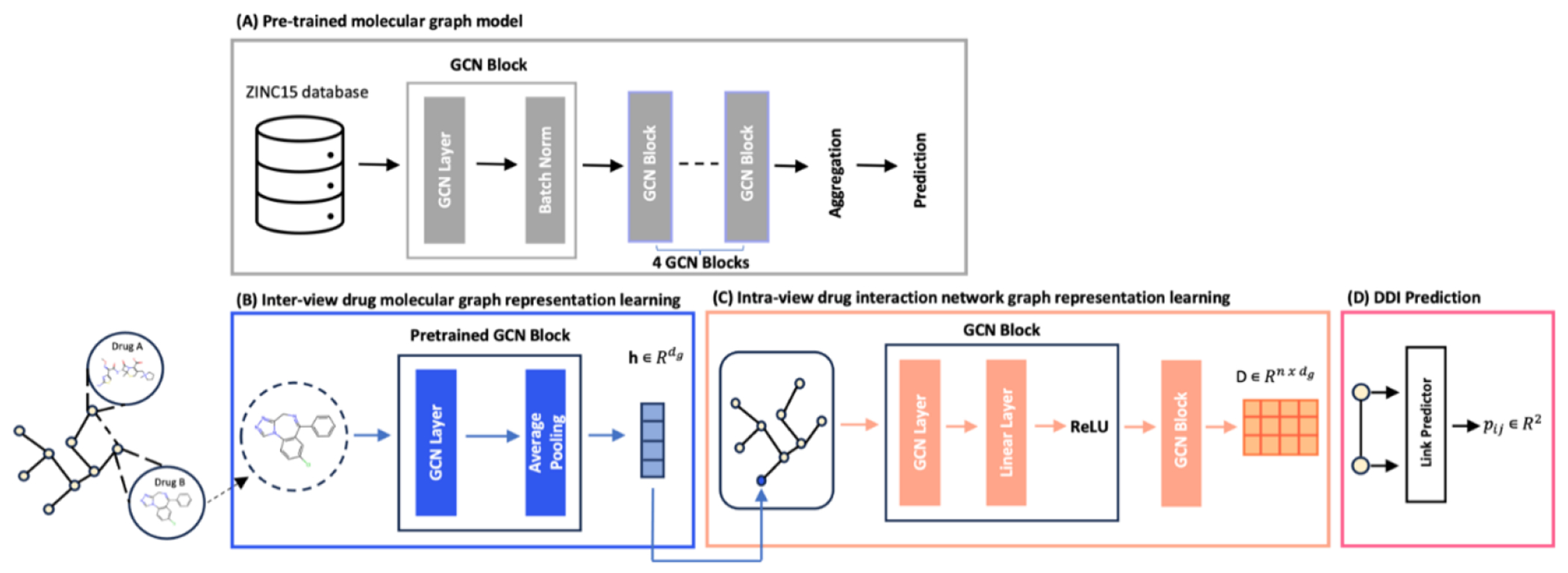
The schematic diagram of our SMG-DDI model.

Our proposed model, SMG-DDI, depicted in Figure 2, is a multi-view graph representation learning framework with four sequential modules. In the initial module, we apply the pre-training method on a Graph Convolution Network (GCN) to acquire transfer learning knowledge of molecules. The second module utilizes the pretrained GCN to encode the inter-view drug molecular graph into embedding vectors. For the third module, handling intra-view graph representation, we integrate information from drug molecular graph embedding and DDI link relationships. The fourth module serves as the link predictor for DDI prediction.

We use RDKit [24] to convert drug molecules from SMILE data into molecular graphs. Our proposed model takes the converted molecular graph’s atom list, chemical bond adjacency matrix, and external DDI network adjacency matrix as input. This section will elaborate on the pre-training strategy, multi-view graph representation learning model architecture, and the model’s objective.

### 2.1. Problem Statement

We use upper letter for matrices (e.g., $A\in R^{m\times n}$), lower letter for vectors (e.g., $h\in R^{d}$), normal characters for scalars (e.g., $d_{g}$ for the dimension of molecule-level embedding), and calligraphic for sets (e.g., *G*).

The DDI prediction task can be defined as a link prediction problem on graph. Let G={V,E} denote a molecular graph with node attribute represents atom as $v_{i}\in V$, and edge attribute represents chemical bond connecting between ith and jth atom as $e_{ij}$ for $\left\{i,j\right\}\in E$, and DDI network $N=\left\{G,L\right\}$ where *L* denotes the interaction links. The task of link prediction is to predict the existence of missing link in network N.

### 2.2. Pre-Trained Molecular Graph Model

**Figure 3.**
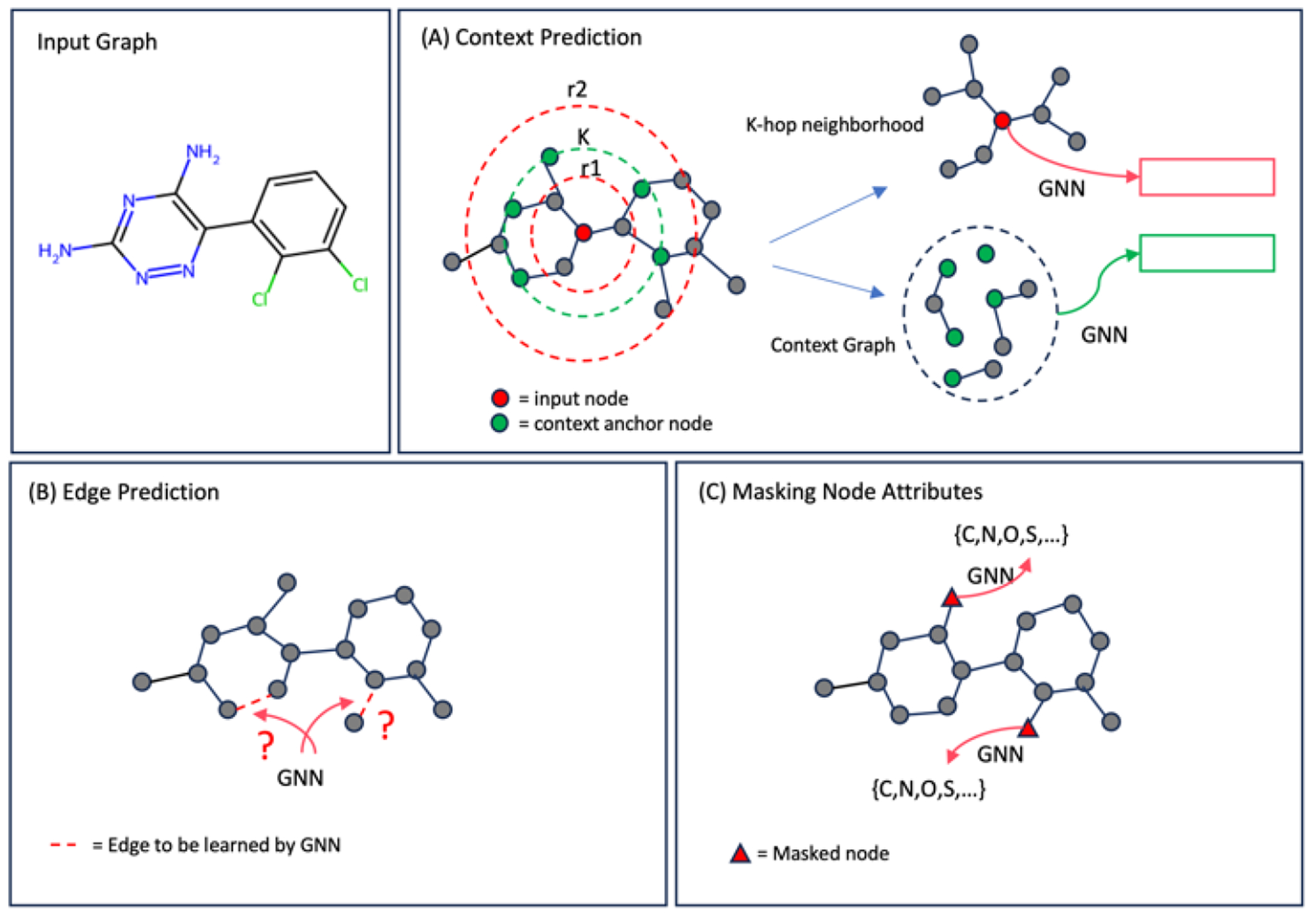
Illustration of pre-training strategy for molecular graph model.

Several key studies demonstrate that pre-training graph models are an effective approach to address the challenge of Out-Of-Distribution (OOD) test sample prediction [25–27]. In this paper, we implement three pre-training methods proposed by Hu et al. [16], which are self-supervised graph representation learning techniques. Our choice of backbone graph model is the GCN, with the aim of gaining transferable molecular graph representation knowledge from a large chemistry dataset. The dataset used for pre-training comprises 2 million unlabeled molecules from the ZINC15 database [28]. Our pretraining models is based on Hu et al. [16] public code (https://github.com/snap-stanford/pretrain-gnns/ (accessed on 18 January 2020)). The training hyperparameters are 100 epochs, learning rate 0.001, batch size 256, 5 layers of GCN. Figure 3 illustrates these three pre-training methods.

- ContextPred: The Context Prediction method, developed by Mikolov et al. [29], utilizes subgraphs to predict the surrounding graph structures. As illustrated in Figure 3A for a molecular graph, an atom has its context graph, encompassing neighborhoods between r1-hops and r2-hops. The main GCN computes the atom’s representation over its context graph, while an auxiliary GCN computes its neighborhood’s representation. The pretrained GCN captures the context of node attributes, mapping nodes with similar structural contexts to their neighbors.
- EdgePred: Hamilton et al. [30] published the method. As depicted in Figure 3B, the Edge Prediction method randomly removes edges from molecular graphs, and the GCN is trained to predict the presence of hidden chemical bonds. The objective of pre-training the GCN with this method is to learn the link attributes between atoms in the molecular graph.
- MaskingNode: In Figure 3C, the Masking Nodes Attributes method, publish by Hu et al. [16], is illustrated in the context of a molecular graph. Similar to pre-training Natural Language Processing models [31], this method randomly masks nodes (atoms) in molecular graphs with special masked tokens. Subsequently, the GCN is applied to obtain corresponding node embeddings and predict the attributes of the masked nodes. Through pre-training, the GCN learns chemistry rules and complex chemical phenomena by capturing the distributions of atoms over the molecular graph.

### 2.3. Multi-View Graph Representation Learning

There are two sequential modules in our proposed model SMG-DDI for the multi-view graph representation learning: (1) the inter-view drug molecular graph representation learning model, and (2) the intra-view drug interaction network representation learning model. The first module encodes drug molecules’ atoms and chemical bond attributes into drug molecular embedding. The second module integrates the drug molecular embedding and the external DDI into the intra-view drug embedding. The architectures of both modules are shown in Figure 2.

### 2.4. Inter-View Drug Molecular Graph Representation Learning

We employ our pretrained GCN as backbone graph model to learn the representation vectors of drug molecular graph. With node attribute matrix V and edge matrix E, the embedding matrix $H\;\in \;R^{n\times d_{g}}$ of drug molecular graph G is formulated as:

(1) $$H=POOL\left({\hat{D}}^{-\frac{1}{2}}\hat{ℰ}{\hat{D}}^{-\frac{1}{2}}𝒱{𝒲}_{p}\right)$$

where *W_p_* is the weight matrix loaded from pretrained GCN; $\hat{ℰ}=ℰ\;+\;I$ is the adjacency matrix with added self-connections; *I* is the identity matrix; $\hat{D}$ is the diagonal node degree matrix of $\hat{ℰ}$ and $\hat{D_{ll}}=\sum_{j}{\hat{ℰ}}_{ij}$; *POOL(·)* is a pooling function use average method.

### 2.5. Intra-View Drug Interaction Network Graph Representation Learning

We build multi-layer GCNs as encoder to learn the intra-view drug interaction network graph representation based on integration of drug molecule attributes and connectivity information over drug interaction graph’s topology. For DDI network N with *n* drugs, drug attribute $H\;\in \;R^{n\times d_{g}}$ and DDI adjacency matrix $L\;\in \;R^{n\times n}$, the DDI network graph embedding $D\;\in \;R^{n\times d_{g}}$ can be derived from:

(2) $$D^{\left(2\right)}=ReLU\left(\hat{ℒ}ReLU\left(\hat{ℒ}HW^{\left(0\right)}\right)W^{\left(1\right)}\right),$$

(3) $$\hat{ℒ}={\tilde{K}}^{-\frac{1}{2}}\left(ℒ\;+\;I_{n}\right){\tilde{K}}^{-\frac{1}{2}},$$

where $D^{\left(2\right)}$ is the drug embedding from 2*nd* layer GCN encoder; $\hat{ℒ}$ is the normalized adjacency matrix from ℒ; $I_{n}$ represents the identity matrix and $\tilde{K}=\sum_{j}{\left(𝒜\;+\;I_{n}\right)}_{ij}$; $W^{\left(0\right)}$ and $W^{\left(1\right)}$ are two trainable weight parameters in 1st and 2nd layer of GCN.

### 2.6. Drug-Drug Interaction Prediction

The last module is designed to predict unknown interaction for the missing links between drugs. We build a link predictor to accomplish this task. For each interaction link $l_{ij}\;\in \;L$ in the DDI network, we first fuse the two drug embedding vectors $d_{i}$ and $d_{j}$ for drug *i* and drug *j* from intra-view graph embedding *D* into the interaction link embedding vector:

(4) $$I_{ij}\;=\;d_{i}\;⨀\;d_{j},$$

where *I* represents the interaction embedding and ⊙ denotes the element-wise product. Then we build two-layer fully connected neural network with interaction link embedding vector *I_ij_* to make the DDI prediction:

(5) $$p_{ij}=\sigma \left(W_{k}ReLU\left(W_{I}I_{ij}\;+\;b_{I}\right)\;+\;b_{k}\right),$$

where *p* ∈ R^2^ is the probability of DDI interaction between drugs *i* and drug *j*; $W_{k},W_{I},b_{I},b_{k}$ are trainable parameters from fully connected neural network.

### 2.7. Objective Function for Model Training

The final objective function contains three parts: (1) the loss between DDI interaction predictions and true labels, (2) the output space matching, which measures the disagreement loss between intra-view and inter-view DDI interaction prediction. To achieve this, we construct an auxiliary drug interaction predictor by passing the interview drug embedding *H* to a fully connected linear layer and a sigmoid function. The prediction from an auxiliary drug interaction predictor for drug i and drug j is denoted as $q_{ij}\;\in \;R^{2}$. (3) the feature space matching, which measures the discrepancy between inter-view and intra-view graph embeddings.

The first loss function is formulated as follows:

(6) $$L_{s}=\sum_{l_{ij}\in ℒ}\left(L_{CE}\left(p_{ij},y_{ij}\right)\;+\;L_{CE}\left(q_{ij},y_{ij}\right)\right),$$

where $y_{ij}$ is the true label of link $l_{ij}$ and $L_{CE}$ is the cross-entropy loss function. The output space matching measurement is formulated as follows:

(7) $$L_{om}=\sum_{l_{ij}\in ℒ}L_{KL}\left(p_{ij},q_{ij}\right),$$

where $L_{KL}$ is the Kullback-Leibler divergence function. For *N* drugs with intra-view graph embedding *d_i_ ∈ D* and inter-view drug molecular embedding *h_i_* ∈ *H* as features. A central moment discrepancy regularizer [23] is employed to match the distributions between inter-view and intra-view graph feature spaces:

(8) $$L_{fm}=\;{\left\|E_{D}-E_{H}\right\|}_{2}+\sum_{k=2}^{K}{\left\|C_{k}^{D}-C_{k}^{H}\right\|}_{2},$$

(9) $$E_{D}\;=\;\frac{1}{N}\sum_{i=1}^{N}d_{i}$$

(10) $$C_{k}^{D}=\frac{1}{N}\sum_{i=1}^{N}{\left(d_{i}\;-\;E_{D}\right)}^{k}$$

where $E_{D}$ the is empirical expectation of the intra-view features, and $C_{k}^{D}$ *is k*-th order central moments of intra-view feature coordinates. $E_{H}$ and $C_{k}^{H}$ are defined in a similar way for inter-view drug molecular graph features. In practice, we compute the central moments up to the fifth order, i.e., *K* = 5. The feature space matching loss $L_{fm}$ enforces the intra-view graph and the inter-view drug molecular graph to have similar feature distributions. Our final loss function for model optimization is formulated as follow:

(11) $$L\;=\;L_{s}\;+\;\alpha L_{om}\;+\;\gamma L_{fm}$$

where α and γ are the hyperparameters that control weights for output space matching loss and feature space matching loss respectively.

## 3. Experiments

### 3.1. Experiment Dataset

Three benchmark public datasets, ZhangDDI [32], ChCh-Miner [33], and DeepDD [17], are used to validate the scalability and robustness of our proposed model. Each dataset’s detailed overview and statistics summary is presented in Table 1.

ZhangDDI is small-scale dataset and contains a relatively small number of drugs. ChCh-Miner is a medium-scale dataset. Compared to ZhangDDI, ChCh-Miner has about three times the number of drugs but the same number of DDI links. DeepDDI is a large-scale dataset with 1694 drugs and 192,284 pairwise DDI links.

The raw data are in SMILES [34] string format. We exclude the data items that cannot be properly converted from SMILES strings into graphs in the data preprocessing step.

### 3.2. Comparing Baseline Methods

Our experimentation aims to illustrate the superiority of our proposed model over baseline methods. The baseline methods encompass two types of graph models: the single-view and hierarchical graph-based models. The single-view graph-based model makes DDI link prediction by learning the node attribute and edge relationship within the molecular graph. The hierarchical graph-based model integrates multi-view information.

- GCN [35]: This approach used a Graph Convolution Network (GCN) for semi-supervised node classification task. Our experimentation uses GCN to encode the drug molecular graphs and make DDI prediction based on molecular graph representation. This is single-view graph-based baseline method.
- GIN [36]: Graph Isomorphism Network (GIN) is second single-view graph-based baseline method in our experimentation. Similar to GCN, we use GIN to make DDI prediction based on its molecular graph representation.
- GraphSAGE [37]: Graph Sample and Aggregation (GraphSAGE) is third single-view graph-based baseline method in our experimentation. It is a inductive representation learning framework, which make DDI link prediction by capturing the structural and contextual information of drugs within the graph.
- SEAL-CI [38]: This is the first approach that applies the hierarchical graph representing learning framework for the node classification tasks. We use this model to extract drug features and learn drug representations to make DDI predictions.
- MIRACLE [11]: This is the state-of-the-art method for DDI prediction tasks. It is a hierarchical graph based model that integrate multi-view graph representation learning by leveraging the bond-aware message passing network (BAMPN) [8] on intra-view molecular graph and GCN inter-view drug-drug relation graph. Furthermore, MIRACLE employed contrastive learning in its framework to conquer over-smoothing problems.

### 3.3. Experimental Settings and Evaluation Metrics

Many DDI prediction applications use the conventional random split method for data splitting. However, the model performance test on conventional random split can be overly optimistic, where test graphs can be structurally different from training graphs. Prior study [19] proves that scaffold data splitting approach splits molecules according to the molecular substructure, providing more realistic estimate of model performance. To validate our model’s out-of-distribution generalization, we use the scaffold data splitting approach to separate our dataset into the train set, validation set, and test set in an 8:1:1 ratio. We conduct experiments five times, each with different random seeds during scaffold data splitting.

Our proposed model comprises the intra-view molecular graph and inter-view drug interaction network graph. The intra-view molecular graph has five layers of GCNs with 300 hidden state dimensions. The inter-view drug interaction network graph has three layers of GCN encoder. Regarding the model training parameters, we set the initial learning rate as 0.001, using the Adam optimize and ReLU activation function. The coefficient α and γ in objective functions are set to 1 and 2, respectively, to achieve the optimal model performance.

Three metrics are chosen to evaluate our proposed model’s effectiveness: Area Under the ROC curve (AUROC), the Area Under the PRC curve (AUPRC), and F1-score. We present the mean and standard deviation of these metrics over five repetitions.

## 4. Results

We assess the effectiveness of our proposed model, SMG-DDI, on three datasets using the scaffold data split setting. In comparison to the baseline models, our proposed model demonstrates superior performance in DDI prediction tasks.

### 4.1. Comparison on the ZhangDDI Dataset

Table 2 presents a model ROC comparison between our proposed model, SMG-DDI, and baseline methods on the ZhangDDI datasets. Three single-view graph-based methods, GCN, GIN, and GraphSAGE, predict DDIs based on pairwise drug representation. However, these single-view graph methods exhibit suboptimal performances as they overlook essential dataset characteristics, such as the drug’s topological structure.

In contrast, two multi-view graph-based methods, SEAL-CI and MIRACLE, which integrate multi-view graphs, outperform the single-view graph methods. SEAL-CI derives drug representation through a continuous graph model but may overlook the graph information equilibrium between different views. On the other hand, MIRACLE utilizes a self-attentive mechanism to generate an inter-view drug representation, focusing on the most significant atoms forming meaningful functional groups in DDI reactions.

**Figure 4.**
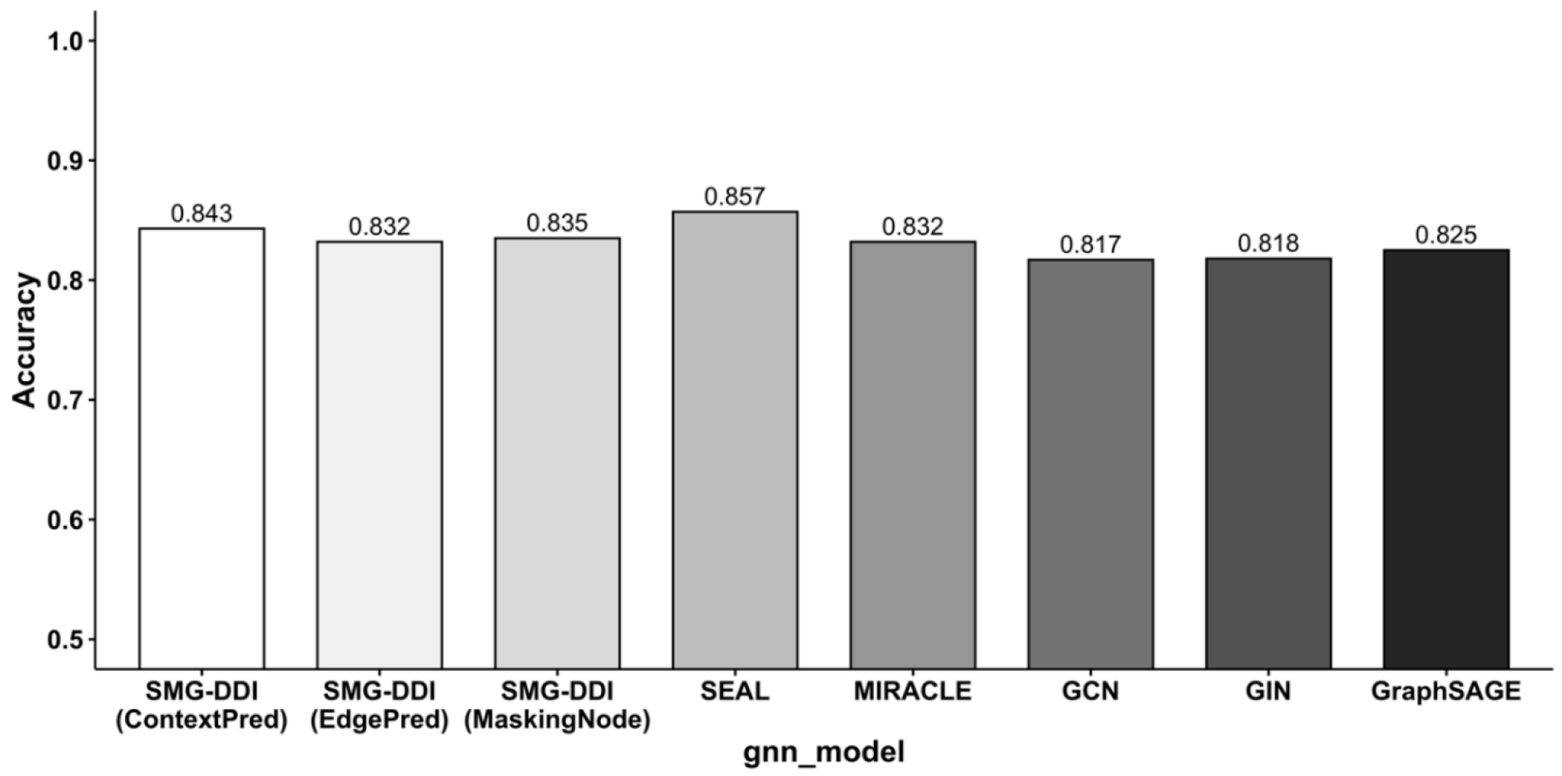
Barplot of accuracy comparisons on ZhangDDI.

Our proposed model, SMG-DDI, is a multi-view graph model leveraging molecular knowledge from related pre-training tasks, achieving comparable performance on the small-scale dataset. Among different pretraining strategies, SMG-DDI with ContextPred demonstrates the best performance compared to state-of-the-art methods. Additionally, the accuracy performances of SMG-DDI with ContextPred and MaskingNode are comparable to SEAL-CI and MIRACLE (Figure 4).

### 4.2. Comparison on the ChCh-Miner Dataset

In Table 3, the experimental results on ChCh-Miner, a medium-scale dataset with few labeled DDI links, are presented. The overall method performances on ChCh-Miner are observed to be lower than those on ZhangDDI. Despite the decrease in prediction performance, multi-view graph-based methods continue to outperform their single-view counterparts. This finding underscores the effectiveness of the multi-view graph-based approach. Notably, MIRACLE excels in learning drug representations even with fewer labeled DDI links, benefits from its graph contrastive learning component.

**Figure 5.**
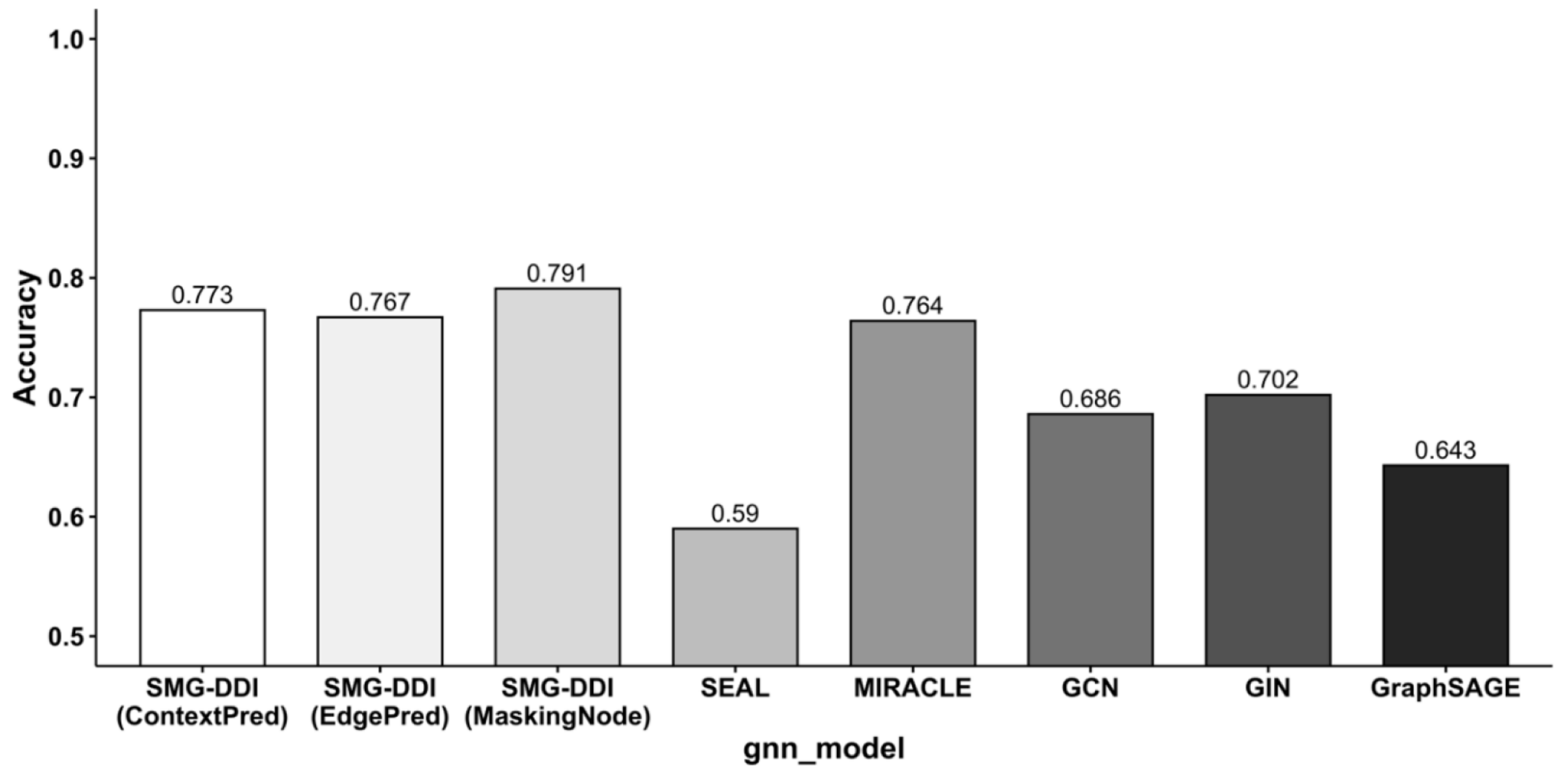
Barplot of accuracy comparisons on ChCh-Miner.

Our proposed SMG-DDI framework employs a feature space matching method to capture underlying chemical patterns in molecular graphs and obtain invariant graph representations. SMG-DDI, pretrained using GCN with three strategies (ContextPred, EdgePred, and Masking Node Attributes), outperforms the baseline multi-view methods. The accuracy performance comparison between the SMG-DDI framework and baseline methods highlights the superiority of our proposed model, particularly on datasets with limited labeled data (Figure 5).

### 4.3. Comparison on the DeepDDI Dataset

**Figure 6.**
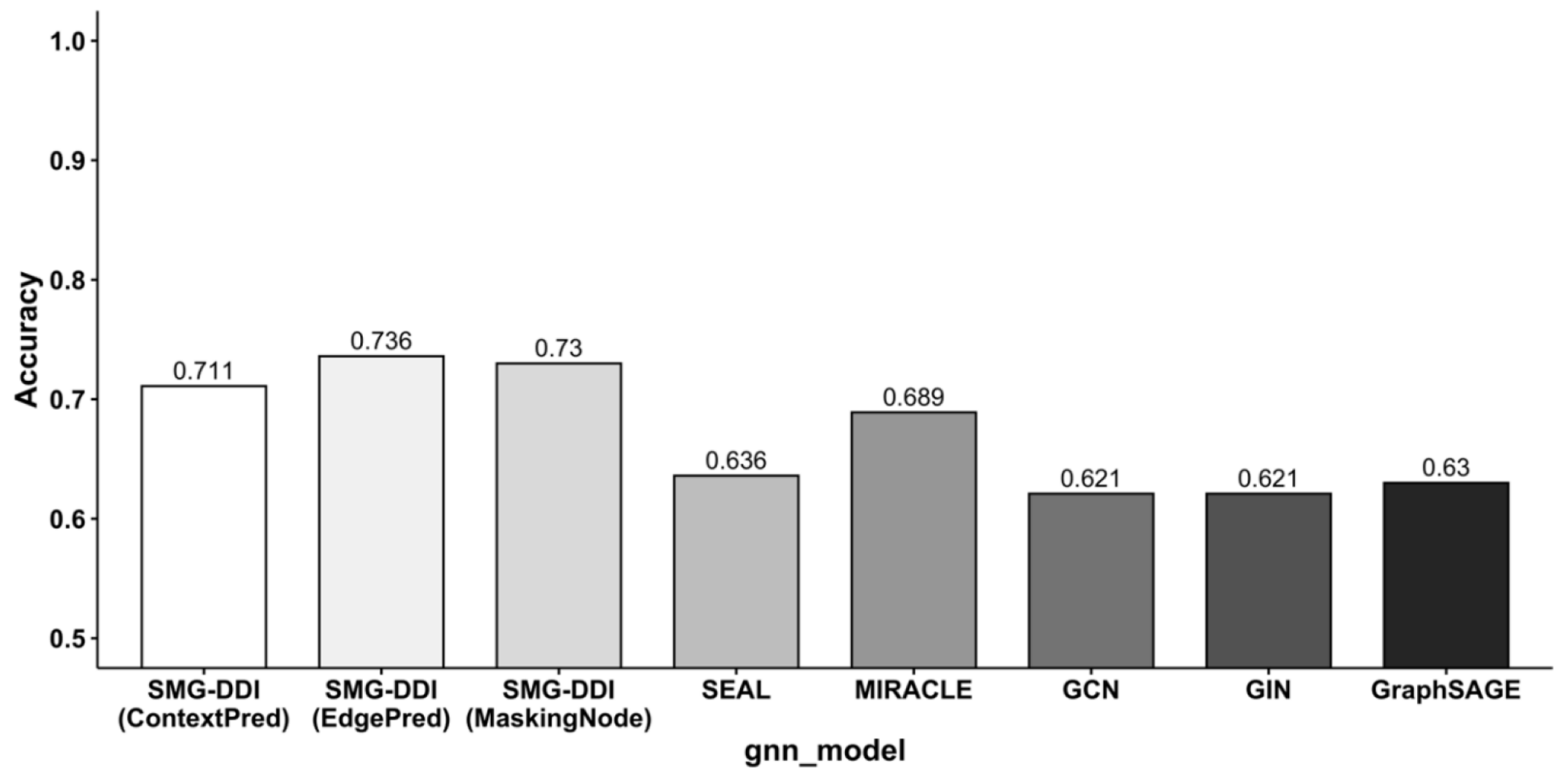
Barplot of accuracy comparisons on DeepDDI.

To assess the robustness of our proposed model, SMG-DDI, we conducted experiments on the DeepDDI dataset, which comprises a large number of labeled DDI instances. The results are presented in Table 4. Figure 6 show the accuracy performance.

Compared to the performance on the ZhangDDI and ChCh-Miner datasets, three single-view graph-based methods exhibited underwhelming results on DeepDDI, highlighting their limitations in handling large variant cases. Notably, SEAL-CI experienced a decrease in DDI prediction performance. In contrast, MIRACLE demonstrated consistent performance across ZhangDDI and ChCh-Miner, showcasing its effectiveness as a robust baseline for DDI prediction tasks, particularly in handling large-scale data.

In terms of pre-training strategy comparisons, both SMG-DDI (EdgePred) and SMG-DDI (MaskingNode) displayed commendable performance on the large-scale DeepDDI dataset. Compared to MIRACLE, SMG-DDI showcased the advantages of leveraging the pretrained graph model. Particularly, SMG-DDI (MaskingNode) demonstrated robustness with superior performance on the ChCh-Miner and DeepDDI datasets. The implementation of MaskingNode pretraining in the graph model retained node attributes corresponding to atom types, facilitating the learning of a molecule’s chemical attributes in the molecular graph. This incorporation of molecular a priori knowledge significantly contributed to enhanced DDI prediction performance.

### 4.4. Ablation Study

**Figure 7.**
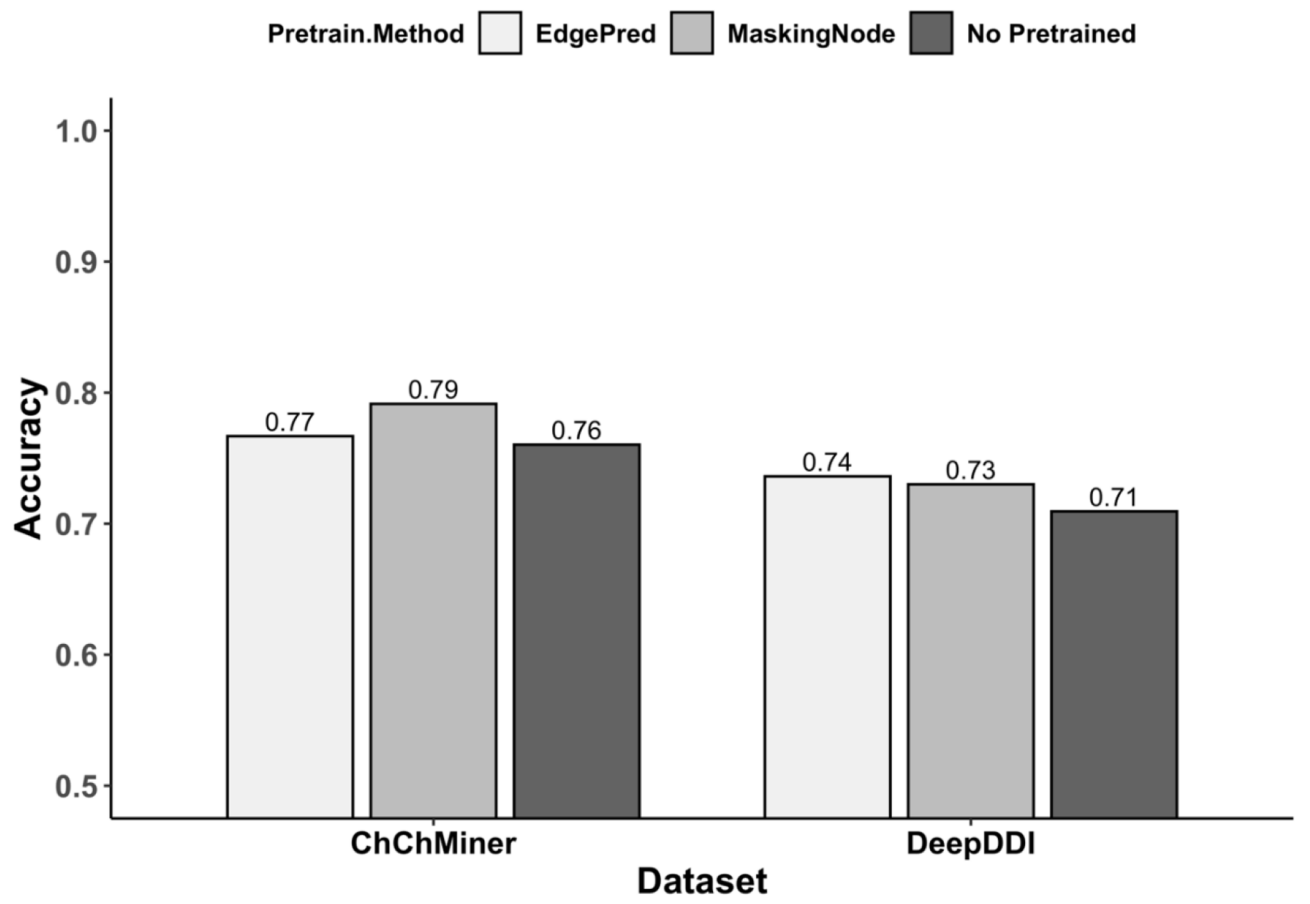
Barplot of accuracy comparisons on ablation experimental.

We performed ablation experiments on the ChCh-Miner and DeepDDI datasets to assess the effectiveness of pretrained graph neural networks. The results, detailed in Table 5 and Figure 7, demonstrate the impact of employing pretrained molecular graph models compared to the same model with random parameter initialization. These experiments confirm that the utilization of pretrained molecular graph models is more effective for DDI prediction tasks.

**Figure 8.**
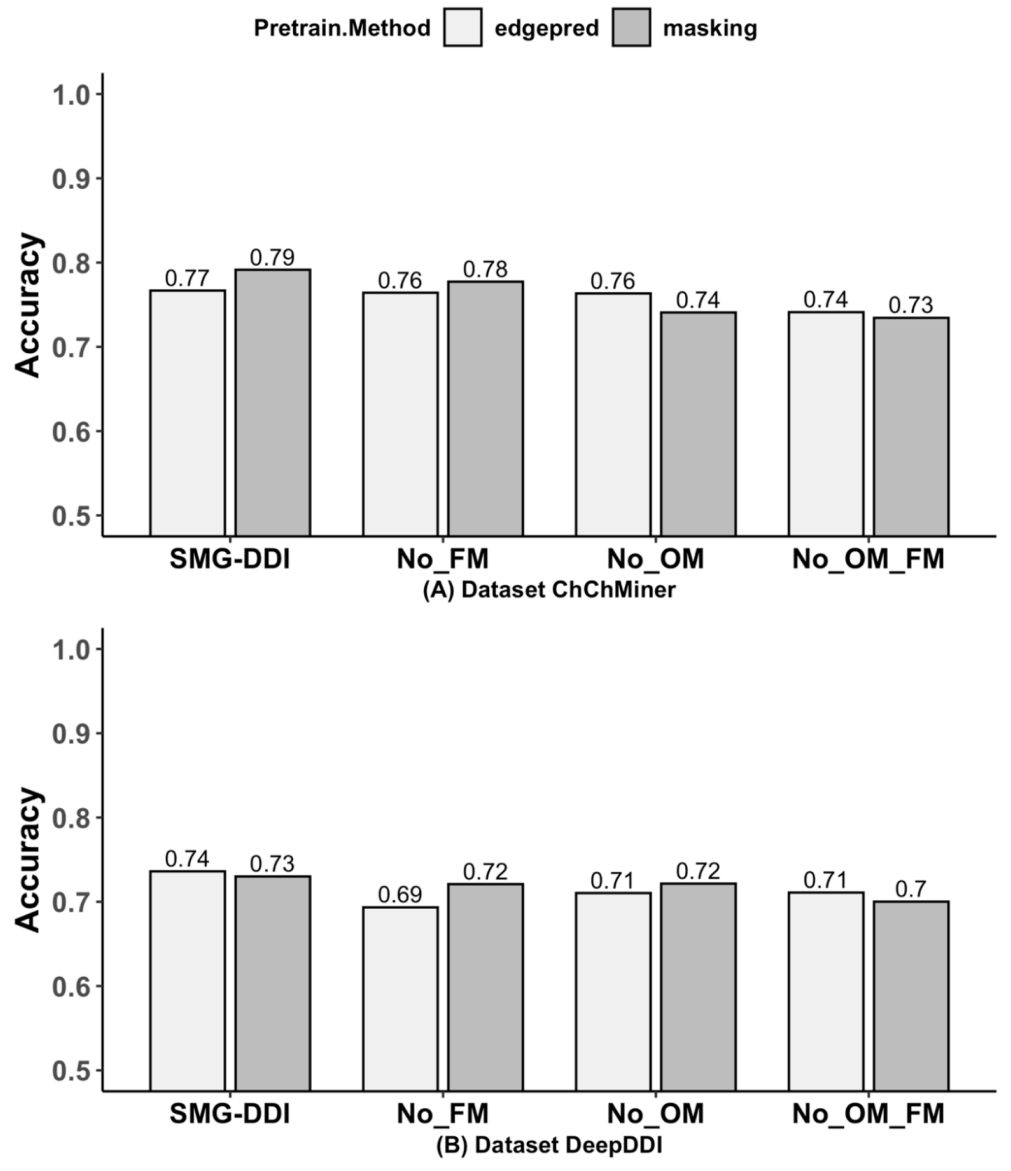
Barplot of accuracy comparisons on ablation experimental of ChChMiner and DeepDDI.

We conducted ablation experiments on the ChCh-Miner and DeepDDI datasets to evaluate the effectiveness of output space matching and feature space matching. The results, presented in Table 6 and Figure 8, highlight the impact of removing output space matching (No_OM), feature space matching (No_FM), and both (No FM & OM). These experiments confirm the importance of using both output space matching and feature space matching. When both are removed, model performance significantly decreases on both datasets. Additionally, the model performs worse when either output space matching or feature space matching is removed compared to the full SMG-DDI model.

### 4.5. Sensitivities of Hyper-Parameters

**Figure 9.**
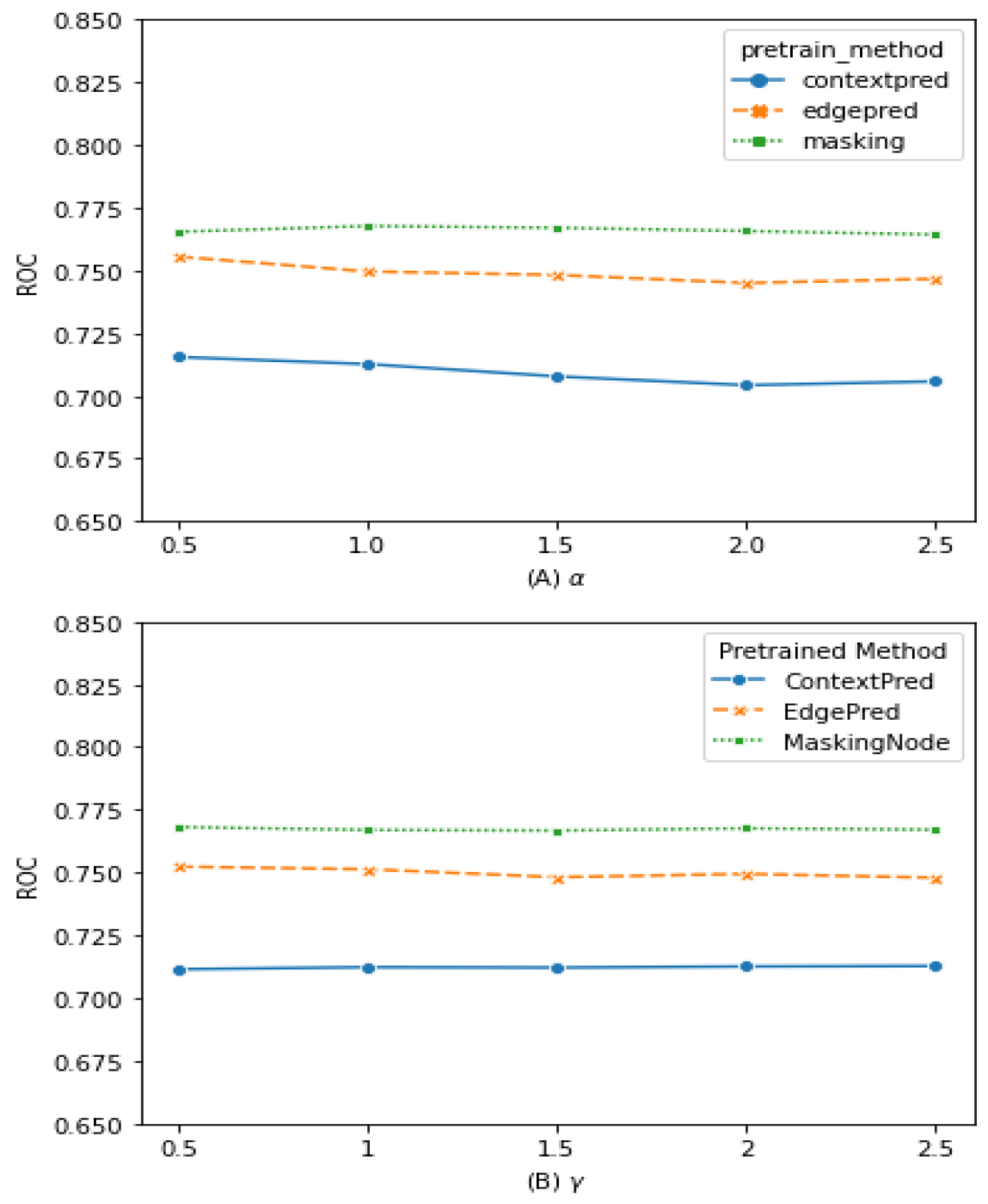
Hyper-parameters Sensitivity.

We also study the influences of different values of hyper-parameters α and γ on the DeepDDI dataset. Hyper-parameters α and γ are coefficient to control the objective function on inter-view and intra-view graph representation training. Figure 9 shows the results by changing one parameter while fixing another one. We first test α in {0.5, 1.0, 1.5, 2, 2.5}, and fix γ=2. We set γ to its optimal value instead of the default value 1. Figure 9A shows our method with three pre-trained models are stable in the test range of α. Next, we test γ in {0.5, 1.0, 1.5, 2, 2.5} with α=1. The γ test result shows in Figure 9B. The overall performance of our method is not sensitive to the values of α and γ.

## 5. Conclusions

In this paper, we introduce a multi-view graph-based model, SMG-DDI which integrates both molecular and drug interaction topological information with the employment of pretrained graph convolution network for drug molecular graph embedding. We employed a central moment discrepancy regularization term to minimizes the distribution discrepancy between multi-view graph representations.

Most of existing hierarchical graph models are experimenting on random splitting datasets. However, the conventional random data splitting approach tends to be overly optimistic and fails to replicate the real-world scenarios. Priori studies, Chen et al. [21] and Sheridan [22], have shown that scaffold data splitting provides a more realistic estimate of model performance in prospective evaluation compared to random data splitting approach. We experiment our model under scaffold data splitting settings on small, median and large size public DDI datasets. We demonstrate our model’s overall performance over baseline models.

We have assessed the efficacy of three pretraining strategies—Context Prediction, Edge Prediction, and Masking Node Prediction—with our proposed model. Our evaluation suggests that all three strategies perform well and produce comparable results on the small-scale ZhangDDI dataset. On the medium-scale ChCh-Miner dataset, the Masking Node strategy appears to achieve slightly better performance than Edge Prediction and demonstrates similar performance to Context Prediction. For the large-scale DeepDDI dataset, both Edge Prediction and Masking Node Prediction prove to be effective and efficient, with Masking Node Prediction achieving higher ROC and AUPRC values, while F1 scores and accuracy remain comparable between the two approaches. Masking Node Prediction works by randomly masking nodes (atoms) in the molecular graphs with special masked tokens, allowing the pretraining GCN to capture chemical rules and complex chemical phenomena by learning the distribution of atoms across the graph. While the three pretraining strategies generally perform similarly, we observe that the Masking Node Prediction, when combined with GCN, output space matching, and feature space matching, offer both superior performance and relative ease of implementation. Our ablation study further suggests that Masking Node Prediction performs slightly better than Edge Prediction when either output space matching or feature space matching is removed. Lastly, our hyper-parameter sensitivity analysis indicates that the model is not highly sensitive to hyper-parameters, particularly on large datasets.

Although our model SMG-DDI achieves good performance on the test datasets. Our model still has limitation that can be improved. We use pretrained graph convolutional network for drug molecular graph embedding. However, we can consider replace our pretrained molecular graph model with NLP-based model to generate molecular representation. MoLFormer-XL [39] is large language model in our scope, which pretrained on 1.1 billion molecules represented as machine-readable strings of text. This model would embed the drug SMILE strings into embeddings, which we could then utilize in a classification model to predict drug-drug interactions.

## Figures and Tables

**Table 1.** Experiment dataset.

Dataset	Number of Drugs	Number of Pairwise DDI Links	Data Size
ZhangDDI	548	48,548	Small
ChCh-Miner	1514	48,514	Medium
DeepDDI	1694	192,284	Large

**Table 2.** Comparative evaluation results on ZhangDDI.

Algorithm	AUROC	AUPRC	F1
GCN	84.89 ± 0.48	84.58 ± 0.52	76.88 ± 0.51
GIN	84.23 ± 0.24	82.12 ± 0.82	75.24 ± 0.26
GraphSAGE	85.14 ± 1.12	84.13 ± 0.25	74.12 ± 0.53
SEAL-CI	90.66 ± 1.3	86.11 ± 2.8	83.99 ± 2.6
MIRACLE	88.91 ± 1.7	87.89 ± 3.9	81.17 ± 3.9
SMG-DDI (ContextPred)	91.34 ± 1.4	90.33 ± 2.6	82.60 ± 3.1
SMG-DDI (EdgePred)	90.64 ± 1.4	90.19 ± 2.7	81.54 ± 3.5
SMG-DDI (MaskingNode)	90.49 ± 1.7	90.04 ± 2.7	81.29 ± 2.9

**Table 3.** Comparative evaluation results on ChCh-Miner.

Algorithm	AUROC	AUPRC	F1
GCN	69.04 ± 2.89	84.22 ± 2.75	79.46 ± 6.24
GIN	70.23 ± 1.64	86.12 ± 1.05	76.82 ± 4.47
GraphSAGE	65.79 ± 2.3	77.43 ± 1.72	78.31 ± 3.76
SEAL-CI	71.52 ± 6.6	82.62 ± 3.1	80.20 ± 9.7
MIRACLE	72.64 ± 3.4	84.21 ± 2.6	82.85 ± 5.2
SMG-DDI (ContextPred)	77.11 ± 6.5	88.19 ± 3.9	83.07 ± 5.7
SMG-DDI (EdgePred)	76.43 ± 4.8	86.77 ± 3.5	82.93 ± 2.6
SMG-DDI (MaskingNode)	78.97 ± 5.0	88.18 ± 4.1	85.11 ± 2.3

**Table 4.** Comparative evaluation results on DeepDDI.

Algorithm	AUROC	AUPRC	F1
GCN	65.45 ± 3.12	72.68 ± 2.49	78.49 ± 3.26
GIN	66.79 ± 1.7	73.24 ± 1.48	79.12 ± 1.25
GraphSAGE	67.01 ± 3.4	73.89 ± 2.13	78.89 ± 3.26
SEAL-CI	67.24 ± 2.8	76.95 ± 3.9	68.35 ± 7.5
MIRACLE	71.53 ± 4.9	78.99 ± 5.0	75.60 ± 4.2
SMG-DDI (ContextPred)	71.26 ± 2.9	78.15 ± 1.4	78.28 ± 2.1
SMG-DDI (EdgePred)	74.95 ± 5.0	81.98 ± 3.5	80.49 ± 2.7
SMG-DDI (MaskingNode)	76.76 ± 2.9	83.32 ± 1.8	79.11 ± 3.0

**Table 5.** Ablation experimental Pretrain GCN vs. No Pretrained GCN.

Dataset	Algorithm	AUROC	AUPRC	F1
**ChCh-Miner**	No Pretrained	73.20 ± 3.9	84.40 ± 3.5	82.87 ± 2.1
**ChCh-Miner**	EdgePred	76.43 ± 4.8	86.77 ± 3.5	82.93 ± 2.6
**ChCh-Miner**	MaskingNode	78.97 ± 5.0	88.18 ± 4.1	85.11 ± 2.3
**DeepDDI**	No Pretrained	72.03 ± 4.0	79.20 ± 5.1	77.78 ± 6.2
**DeepDDI**	EdgePred	74.95 ± 5.0	81.98 ± 3.5	80.49 ± 2.7
**DeepDDI**	MaskingNode	76.76 ± 2.9	83.32 ± 1.8	79.11 ± 3.0

**Table 6.** Ablation experimental Output Space Matching (OM) and Feature Space Matching (FM).

Dataset	Space Matching	Algorithm	AUROC	AUPRC	F1
**ChCh-Miner**	No FM	EdgePred	74.85 ± 1.0	84.76 ± 4.8	84.80 ± 3.6
**ChCh-Miner**	No FM	MaskingNode	74.69 ± 3.0	85.09 ± 1.8	85.90 ± 3.7
**ChCh-Miner**	No OM	EdgePred	72.51 ± 8.2	84.13 ± 4.8	82.99 ± 3.6
**ChCh-Miner**	No OM	MaskingNode	72.05 ± 10.5	83.27 ± 5.9	81.29 ± 4.2
**ChCh-Miner**	No FM & OM	EdgePred	72.11 ± 4.4	84.23 ± 2.3	80.95 ± 3.9
**ChCh-Miner**	No FM & OM	MaskingNode	72.69 ± 3.2	84.75 ± 2.8	79.28 ± 9.3
**ChCh-Miner**	FM & OM	EdgePred	76.43 ± 4.8	86.77 ± 3.5	82.93 ± 2.6
**ChCh-Miner**	FM & OM	MaskingNode	78.97 ± 5.0	88.18 ± 4.1	85.11 ± 2.3
**DeepDDI**	No FM	EdgePred	73.86 ± 2.7	81.99 ± 2.5	75.48 ± 5.1
**DeepDDI**	No FM	MaskingNode	75.51 ± 2.7	80.90 ± 2.1	78.55 ± 1.3
**DeepDDI**	No OM	EdgePred	73.23 ± 3.4	81.09 ± 2.2	79.03 ± 2.2
**DeepDDI**	No OM	MaskingNode	74.15 ± 3.3	81.88 ± 2.0	78.65 ± 2.5
**DeepDDI**	No FM & OM	EdgePred	73.37 ± 2.3	80.05 ±2.6	77.50 ± 4.5
**DeepDDI**	No FM & OM	MaskingNode	73.11 ± 2.6	81.15 ± 1.4	76.28 ± 4.1
**DeepDDI**	FM & OM	EdgePred	74.95 ± 5.0	81.98 ± 3.5	80.49 ± 2.7
**DeepDDI**	FM & OM	MaskingNode	76.76 ± 2.9	83.32 ± 1.8	79.11 ± 3.0

## Data Availability

Source codes and data are available at https://github.com/dukekuang/SMG_DDI (accessed on 19 September 2024).
